# Predictors of quality of life among adolescents and young adults with a bleeding disorder

**DOI:** 10.1186/s12955-017-0643-7

**Published:** 2017-04-07

**Authors:** John M. McLaughlin, James E. Munn, Terry L. Anderson, Angela Lambing, Bartholomew Tortella, Michelle L. Witkop

**Affiliations:** 1 Pfizer Inc, Global Innovative Pharma, 500 Arcola Rd, Collegeville, PA 19426 USA; 2 University of Michigan Hemophilia Treatment Center, 1500 E. Medical Center Drive, Ann Arbor, MI USA; 3 Henry Ford Hemophilia & Thrombosis Treatment Center, 2799 W Grand Blvd, Detroit, MI USA; 4 Northern Regional Bleeding Disorder Center, 1105 Sixth St, Traverse City, MI USA; 5 Currently: Bayer HealthCare, Whippany, NJ USA

**Keywords:** Chronic pain, Hemophilia, Pain management, Patient adherence, Prophylaxis, von Willebrand disease

## Abstract

**Background:**

Health-related quality of life (HRQoL) in adolescents and young adults with bleeding disorders is under-researched. We aimed to describe factors related to HRQoL in adolescents and young adults with hemophilia A or B or von Willebrand disease.

**Methods:**

A convenience sample of volunteers aged 13 to 25 years with hemophilia or von Willebrand disease completed a cross-sectional survey that assessed Physical (PCS) and Mental (MCS) Component Summary scores on the SF-36 questionnaire. Quantile regression models were used to assess factors associated with HRQoL.

**Results:**

Of 108 respondents, 79, 7, and 14% had hemophilia A, hemophilia B, and von Willebrand disease, respectively. Most had severe disease (71%), had never developed an inhibitor (65%), and were treated prophylactically (68%). Half of patients were aged 13 to 17 years and most were white (80%) and non-Hispanic (89%). Chronic pain was reported as moderate to severe by 31% of respondents. Median PCS and MCS were 81.3 and 75.5, respectively. Quantile regression showed that the median PCS for women (61% with von Willebrand disease) was 13.1 (95% CI: 2.4, 23.8; *p* = 0.02) points lower than men. Ever developing an inhibitor (vs never) was associated with a 13.1-point (95% CI: 4.7, 21.5; *p* < 0.01) PCS reduction. MCS was 10.0 points (95% CI: 0.7, 19.3; *p* = 0.04) higher for prophylactic infusers versus those using on-demand treatment. Compared with patients with no to mild chronic pain, those with moderate to severe chronic pain had 25.5-point (95% CI: 17.2, 33.8; *p* < 0.001) and 10.0-point (95% CI: 0.8, 19.2; *p* = 0.03) reductions in median PCS and MCS, respectively.

**Conclusions:**

Efforts should be made to prevent and manage chronic pain, which was strongly related to physical and mental HRQoL, in adolescents and young adults with hemophilia and von Willebrand disease. Previous research suggests that better clotting factor adherence may be associated with less chronic pain.

## Background

Therapeutic advancements [1–4], improved treatment approaches [5–8], and enhanced care delivery models [9–12] have resulted in highly active lifestyles and near-normal life expectancy for persons diagnosed with a bleeding disorder [13]. As treatments continue to improve, patient care will continue to evolve toward preventing bleeding disorder complications, such as hemophilic arthropathy, chronic pain, and inhibitor development—with the ultimate goal of attaining and maintaining high health-related quality of life (HRQoL) for those affected by the disorder [14]. Previous research has shown that among persons with a bleeding disorder, HRQoL may be related to pain, treatment type (prophylaxis vs on-demand), adherence, age-related concerns, inhibitor status, or depression [14–21]. These findings suggest that bleeding disorders particularly diminish the physical aspects of HRQoL and that alleviating the clinical burden of bleeding disorders may be particularly important for improving and maintaining physical well-being and overall HRQoL.

Measuring HRQoL is a unique way to assess the general well-being and perceived health of individuals living with a bleeding disorder, the experiences related to having a bleeding disorder, and satisfaction with treatment [22]. However, very little, if any, data exist that describe HRQoL among adolescent and young adult (AYA) persons diagnosed with a bleeding disorder. AYAs are a unique population of bleeding disorder patients who are often just starting to take more responsibility for the management of their own disease and developing treatment habits that can carry over into adult life [23]. Entering adolescence and young adulthood might mean finally confronting the demands that managing a bleeding disorder entails. The aim of this study was to investigate the relationships between pain management, adherence to prescribed treatment, and HRQoL in AYA persons with hemophilia (PWH) or von Willebrand disease (VWD).

## Methods

### Study population and recruitment

Data describing HRQoL, adherence to prescribed treatment regimens, and level of chronic pain among AYA PWH or VWD were obtained as part of the larger Interrelationship Between Management of Pain, Adherence to Clotting-factor Treatment, and Quality of Life (IMPACT QoL) study. As the name suggests, the IMPACT QoL study had the primary goal of assessing the relationship between validated measures of pain, clotting-factor adherence, and HRQoL among AYA PWH or VWD. Data describing the relationship between adherence to a prescribed clotting factor treatment regimen and chronic pain, along with racial differences in chronic pain and QoL from this study, were previously reported [24, 25]. Data were collected via a one-time, cross-sectional, online survey from a convenience sample of AYA PWH or VWD. To be eligible to complete the survey, participants had to be aged 13 to 25 years, read, write, and speak English, and have hemophilia A, hemophilia B, or VWD. Recruitment occurred at major US hemophilia meetings (e.g., Inhibitor Summits and National Hemophilia Foundation meetings), US hemophilia treatment centers, and through a Facebook^©^ page dedicated to the study from April through December of 2012. All surveys were completed electronically using SurveyMonkey® and Apple® iPads®. Informed consent was obtained from all individual participants included in the study. The study was approved by the Munson Medical Center (Traverse City, MI) institutional review board prior to data collection. All data were de-identified prior to analysis. The current study uses the IMPACT QoL survey data to determine factors associated with better HRQoL among AYA (aged 13–25 years) PWH or VWD.

### Measurement

HRQoL was measured using the 36-Item Short Form Health Survey (SF-36). The SF-36 is composed of 8 multi-item scales (35 items) assessing the following: (1) bodily pain (BP, 2 items), (2) physical function (PF, 10 items), (3) role limitations due to physical health problems (RP, 4 items), (4) general health (GH, 5 items), (5) vitality (VT, 4 items), (6) social functioning (SF, 2 items), and (7) role limitations due to emotional problems (RE, 3 items) and emotional well-being/mental health (MH, 5 items) [26]. The 36th item, which asks about health change, is not included in the scale or summary scores. Each subscale is calculated by taking the average of the participant’s responses to the questions contained in the subscale and then standardizing it so that each had a final range of 0 (lowest level of functioning) to 100 (highest level of functioning). These eight scales can be aggregated into two summary measures: the Physical (PCS) and Mental (MCS) Component Summary scores, where higher scores represent better health. The scoring algorithm for PCS includes positive weights for the PF, RP, BP, GH, and VT scales and negative weights for the SF, RE, and MH. The scoring algorithm for MCS includes positive weights for the VT, SF, RE, and MH scales and negative weights for the PF, RP, BP, and GH scales. Three scales (PF, RP, and BP) correlate most highly with the physical component and contribute most to the scoring of the PCS. The MCS correlates most highly with the MH, RE, and SF scales, which also contribute most to the scoring of the MCS measure. Three of the scales (VT, GH, and SF) have noteworthy correlations with both components. The SF-36 was constructed for self-administration by persons 14 years of age and older, and for administration by a trained interviewer in person or by telephone [26].

Adherence was assessed using the Validated Hemophilia Regimen Treatment Adherence Scale (VERITAS)-Pro [27] and VERITAS-PRN [28] for prophylactic and on-demand (i.e., episodic) participants, respectively. VERITAS scores range from 24 (most adherent) to 120 (least adherent). As an experimental measure, we also combined VERITAS-Pro and VERITAS-PRN responses into one category (VERITAS-combined) [25] to evaluate the relationship between adherence and HRQoL for both prophylactic and on-demand AYA PWH simultaneously. The cutoff for non-adherent prophylactic participants was a total VERITAS-Pro score ≥57 as previously established [27]. The cutoff for non-adherent on-demand patients has not been previously described and was defined as those with VERITAS-PRN in the highest quartile of all responses. This value was chosen because the VERITAS-Pro cutoff was approximately the 75th percentile of all responses.

Chronic pain was measured using the revised Faces Pain Scale-Revised (FPS-R). The FPS-R is a visual scale composed of six faces illustrating an increasing level of pain intensity. Respondents were asked to choose the face that best describes the intensity of the chronic pain they experienced. In the IMPACT HRQoL survey, chronic pain was defined as ‘pain that you have every day or almost every day, and that always or almost always seems to be there even when you are not having a bleed at that moment.’ FPS-R scores range from 0 to 10 with the faces representing the lowest and highest levels of pain intensity coded as 0 and 10, respectively. The FPS-R is highly correlated with the visual analog scale (r = .93) and with the colored analog scale (r = .84), demonstrating strong validity. Reliability and validity of the FPS-R have been established for a broad age range, ranging from children as young as 4 years old to adults [29]. For purpose of analysis, chronic pain was dichotomized as *high* for those who reported their pain as ‘moderate,’ ‘severe,’ ‘very severe,’ or ‘worst pain possible’ (i.e., FPS-R ≥4) and *low* for ‘mild pain’ or ‘no pain’ (i.e., FPS-R <4).

Other self-reported data collected included information about participant age, gender, race, ethnicity, health insurance status/type, and the educational level of the participants’ parents. Data were also collected about bleeding disorder type (hemophilia A or B, or VWD), whether or not the participant ever developed an inhibitor to treatment, and bleeding disorder severity. For hemophilia A and B, severity was classified as mild, moderate, or severe corresponding to 6 to 50%, 1 to 5%, and <1%, respectively, of the normal amount of clotting factor. VWD was classified as mild, moderate, or severe corresponding to Type I (lower than normal levels of von Willebrand factor), Type II (lower than normal levels and improper functioning of von Willebrand factor), and Type III disease (absence of von Willebrand factor in the blood).

### Statistical analysis

Because of their skewed nature, descriptive statistics and univariate relationships were assessed by tabulating median PCS and MCS SF-36 scores with interquartile ranges (IQRs) by participant sociodemographic and clinical characteristics. Percentages were used to describe categorical variables and statistical association with SF-36 scores was assessed using the non-parametric Wilcoxon rank sum test or Kruskal Wallis test (depending on degrees of freedom), as they do not assume a normal distribution of the residuals. As part of an exploratory analysis, we also examined univariate relationships between participant sociodemographic and clinical characteristics and each of the eight SF-36 subscales. Because this portion of the analysis was only exploratory, no adjustments for multiple comparisons were made.

The primary outcome variables of interest (SF-36 PCS and MCS scores) were largely skewed, thus multivariable, quantile regression models were used to assess factors associated with physical and emotional SF-36 subscales. Factors assessed for their relationship with HRQoL included: age, gender, race and ethnicity, parent’s education level, bleeding disorder type and severity, history of inhibitor development, level of chronic pain, clotting-factor treatment adherence, and treatment regimen type (on-demand vs prophylactic). Due to the large number of variables collected as part of the survey and because of the small sample size inherent in rare disease research, in addition to the fully adjusted models, final parsimonious models were constructed. In the final parsimonious models, we decided, a priori, to include covariates in the model only if they (1) were statistically significant at a two-tailed alpha level of .05, (2) changed the coefficient of another statistically significant model parameter by at least 10% to 15% (i.e., confounded) [30], or (3) improved the precision of another statistically significant parameter already in the model. All statistical analyses were performed using SAS 9.2 (SAS Institute, Inc; Cary, NC). All *p*-values were calculated using two-sided tests.

## Results

Respondent characteristics are summarized in Table 1. Overall, 108 AYAs with hemophilia A, hemophilia B, and VWD participated; half of the participants were aged 13 to 17 years, and the majority were male (83%). Male participants were more likely to have hemophilia vs VWD (96 vs 4%), while the opposite was true for female participants (39 vs 61%). The majority (94%) of respondents had some type of health insurance.

**Table 1:** Respondent characteristics (*n* = 108)

Characteristic	*Number*	*Percent*
Age, years		
13–17	54	50
18–25	54	50
Gender		
Male	90	83
Female	18	17
Race		
White	86	80
Non-white^a^	22	20
Ethnicity		
Hispanic	12	11
Non-Hispanic	96	89
Health insurance^b^		
Medicaid or VA only^c^	34	32
Commercial only	46	43
Both	8	7
Insured – type unknown	12	11
Uninsured	6	6
Mother’s education level		
Bachelor’s degree or higher	38	35
Less than Bachelor’s degree	70	65
Father’s education level		
Bachelor’s degree or higher	31	29
Less than Bachelor’s degree	71	71
Bleeding disorder		
Hemophilia A	85	79
Hemophilia B	5	7
von Willebrand disease	15	14
Severity		
Mild	22	20
Moderate	9	8
Severe	77	71
Inhibitor development		
Ever	38	35
Never	70	65
Treatment regimen		
On-demand	35	32
Prophylaxis	73	68
Chronic pain^d^		
None to mild	74	69
Moderate to severe	34	31
Clotting factor adherence^e^		
Adherent	77	71
Non-adherent	31	29

^a^Most (73%) of non-white respondents were black or African American, 14% were mixed race, 9% were Asian, and 5% were American Indian or Alaskan Native

^b^n = 78, two respondents answered ‘Don’t know’ as to whether or not they had health insurance, and were not included

^c^Only two respondents had VA only insurance, the others had Medicaid only

^d^Chronic pain was measured using the revised Faces Pain Scale (FPS-R) and was dichotomized as FPS-R < 4 (i.e., ‘mild’ or ‘no pain) and FPS-R ≥4 (ie, ‘moderate’ to ‘worst pain possible’)

^e^Adherence was assessed using the Validated Hemophilia Regimen Treatment Adherence Scale (VERITAS)-Pro and VERITAS-PRN for prophylactic and on-demand participants, respectively. The cutoff for non-adherent prophylactic participants was a total VERITAS-Pro score ≥57 as established in previously by Duncan and colleagues [25]. This value was chosen because the VERITAS-Pro cutoff was approximately the 75th percentile of all responses

**Fig. 1:**
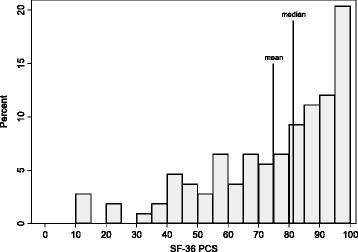
Distribution of SF-36 Physical Component Summary scores (*n* = 108). SF-36, 36-Item Short Form Health Survey; PCS, Physical Component Summary

**Fig. 2:**
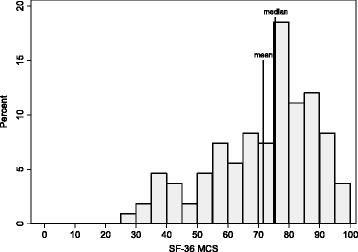
Distribution of SF-36 Mental Component Summary scores (*n* = 108). SF-36, 36-Item Short Form Health Survey; MCS, Mental Component Summary

**Fig. 3:**
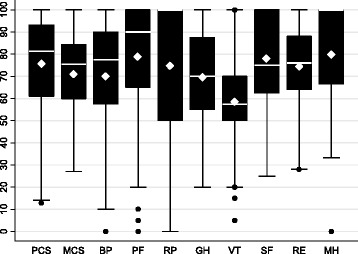
Box plots for SF-36 component and subscale scores (*n* = 108). The solid white line represents median value, and the white diamond represents the mean value. PCS, Physical Component Summary score; MCS, Mental Component Summary; BP, bodily pain; PF, physical function; RP, role limitations due to physical health problems; GH, general health; VT, vitality; SF, social functioning; RE, role limitations due to emotional problems; MH, emotional well-being/mental health

Median PCS and MCS were 81.3 (IQR: 61.1–93.1; range: 12.9–100) and 75.5 (IQR: 60.0–84.3.1; range: 27.1–100), respectively (Figs. 1 and 2). Mean values for PCS, MCS, and the eight multi-item subscales were generally lower than the median (with the exception of VT and SF) due to low outlying values (Fig. 3). At the univariate level, young adults (vs adolescents), non-whites, those who reported ever developing an inhibitor, and those who reported moderate to severe (vs none to mild) chronic pain had statistically significantly lower PCS scores (Table 2). Young adults (vs adolescents), those who reported moderate to severe (vs none to mild) chronic pain, and those who were non-adherent to prescribed clotting-factor treatment regimens had statistically significantly lower MCS scores (Table 2). Univariate level differences for PCS, MCS, and the eight multi-item subscales by respondent characteristic are shown in the supplement (Figs 4 and 5 for median and mean values, respectively).

**Table 2:** Median (IQR) SF-36 Physical and Mental Component Summary scores by respondent characteristic (*n* = 108)

Characteristic	SF-36 PCS	*p*-value	SF-36 MCS	*p*-value
Age		0.03		0.04
13–17	86.3 (64.8, 95.7)		79.5 (64.6, 85.7)	
18–25	75.1 (59.0, 87.1)		73.4 (58.6, 78.9)	
Gender		0.12		0.10
Male	83.0 (59.0, 94.5)		76.8 (59.6, 85.7)	
Female	78.9 (64.8, 86.0)		71.4 (64.6, 76.4)	
Race		0.02		0.94
White	83.6 (65.7, 94.3)		75.9 (59.6, 84.3)	
Non-white^a^	62.6 (48.3, 84.8)		74.3 (64.6, 85.7)	
Ethnicity		0.18		0.73
Hispanic	71.9 (59.3, 81.5)		77.0 (67.0, 82.1)	
Non-Hispanic	83.0 (61.1, 94.2)		75.2 (59.6, 84.8)	
Health insurance^b^		0.13		0.21
Medicaid or VA only^c^	79.4 (56.0, 86.4)		75.9 (64.6, 85.7)	
Commercial only	86.2 (65.0, 95.7)		78.8 (65.0, 84.3)	
Both	71.0 (60.8, 77.7)		72.9 (59.3, 81.3)	
Insured, type unknown	83.7 (69.6, 91.8)		64.3 (54.6, 86.3)	
Uninsured	63.0 (41.4, 90.5)		61.8 (42.9, 69/3)	
Mother’s education level		0.19		0.71
Bachelor’s degree or higher	86.1 (65.7, 95.5)		75.7 (60.4, 85.4)	
Less than Bachelor’s degree	80.1 (58.1, 91.0)		75.5 (59.6, 84.3)	
Father’s education level		0.10		0.46
Bachelor’s degree or higher	88.3 (67.4, 95.5)		76.4 (58.6, 87.1)	
Less than Bachelor’s degree	79.8 (58.1, 91.0)		75.4 (60.4, 82.9)	
Bleeding disorder		0.78		0.34
Hemophilia A	81.2 (58.1, 94.5)		76.4 (59.3, 85.7)	
Hemophilia B	81.7 (73.2, 88.5)		76.1 (65.0, 81.8)	
von Willebrand	80.5 (64.8, 86.2)		70.0 (64.6, 76.4)	
Severity		0.98		0.37
Mild	81.6 (65.0, 91.7)		73.2 (61.8, 78.6)	
Moderate	86.0 (64.0, 88.6)		75.4 (60.4, 87.1)	
Severe	79.8 (58.1, 94.3)		76.4 (59.6, 85.7)	
Inhibitor development		<0.01		0.16
Ever	69.0 (45.0, 85.2)		73.4 (56.8, 83.9)	
Never	86.1 (67.9, 94.5)		78.2 (64.6, 86.4)	
Treatment regimen		0.99		0.13
On-demand	81.9 (66.2, 91.7)		72.9 (59.3, 78.9)	
Prophylaxis	81.2 (58.1, 94.5)		77.1 (60.4, 85.7)	
Chronic pain^d^		<0.0001		0.01
None to mild	87.1 (75.5, 95.5)		77.1 (64.6, 86.8)	
Moderate to severe	59.0 (45.0, 74.5)		72.9 (56.8, 78.8)	
Clotting factor adherence^e^		0.24		0.04
Adherent	82.6 (64.8, 95.5)		76.4 (64.6, 84.3)	
Non-adherent	79.0 (55.5, 90.5)		68.2 (48.6, 78.6)	

^a^Most (73%) of non-white respondents were black or African American, 14% were mixed race, 9% were Asian, and 5% were American Indian or Alaskan Native

^b^ *n* = 78, two participants answered ‘Don’t Know’ to whether or not they had health insurance and were not included

^c^Only two participants had VA only insurance, the others had Medicaid only

^d^Chronic Pain was measured using the revised Faces Pain Scale (FPS-R) and was dichotomized as FPS-R < 4 (i.e., ‘mild’ or ‘no pain) and FPS-R ≥ 4 (i.e., ‘moderate’ to ‘worst pain possible’)

^e^Adherence was assessed using the Validated Hemophilia Regimen Treatment Adherence Scale (VERITAS)-Pro and VERITAS-PRN for prophylactic and on-demand participants, respectively. The cutoff for non-adherent prophylactic participants was a total VERITAS-Pro score ≥57, as established previously by Duncan and colleagues [25]. This value was chosen because the VERITAS-Pro cutoff was approximately the 75th percentile of all responses

*SF-36* 36-Item Short Form Health Survey, *MCS* Mental Component Summary, *PCS* Physical Component Summary

Final quantile regression models suggested that, compared with men, median PCS for women was 13.1 (95% CI: 2.4, 23.8; *p* = 0.02) points lower. Ever developing an inhibitor (vs never) was significantly associated with a 13.1 (95% CI: 4.7, 21.5; *p* < 0.01) point reduction in PCS. Prophylactic infusers (vs on-demand) had a 10.0 (95% CI: 0.7, 19.3; *p* = 0.04) point increase in median MCS. Compared with those with no or mild chronic pain, those with moderate to severe chronic pain had a 25.5 (95% CI: 17.2, 33.8; *p* < 0.001) and 10.0 (95% CI: 0.8, 19.2; *p* = 0.03) point reduction in median PCS and MCS, respectively. Tables 3 and 4 show final quantile regression models modeling PCS and MCS scores, respectively.

**Table 3:** Quantile (median) regression model estimating median SF-36 Physical Component Summary scores, 2012 (*n* = 108)

Characteristic	Coefficient (95% CI)	*p*-value
Gender		0.02
Female	−13.1 (−23.8, −2.4)	
Male	reference	
Inhibitor development		<0.01
Ever	−13.1 (−21.5, −4.7)	
Never	reference	
Chronic pain^a^		<0.001
Moderate to severe	−25.5 (−33.8, −17.2)	
None to mild	reference	

^a^Chronic pain was measured using the revised Faces Pain Scale (FPS-R) and was dichotomized as FPS-R <4 (i.e., ‘mild’ or ‘no pain) and FPS-R ≥4 (i.e., ‘moderate’ to ‘worst pain possible’)

**Table 4:** Quantile (median) regression model estimating median SF-36 Mental Component Summary scores, 2012 (*n* = 108)^a^

Characteristic	Coefficient (95% CI)	*p*-value
Treatment regimen		0.04
Prophylaxis	10.0 (0.7, 19.3)	
On-demand	reference	
Chronic pain^b^		0.03
Moderate to severe	−10.0 (−19.2, −0.8)	
None to mild	reference	

^a^Ethnicity (Hispanic *vs* non-Hispanic) and history of inhibitor development (ever *vs* never) were also included in the model, though not statistically significant, because they increased the precision of the estimates

^b^Chronic pain was measured using the revised Faces Pain Scale (FPS-R) and was dichotomized as FPS-R < 4 (i.e., ‘mild’ or ‘no pain) and FPS-R ≥ 4 (i.e., ‘moderate’ to ‘worst pain possible’)

*CI* confidence interval

## Discussion

Evaluating HRQoL will be required to determine the effectiveness of care strategies and to measure the impact of living with a bleeding disorder over time. To our knowledge, limited data are available regarding the HRQoL of AYAs with hemophilia. In contrast to previous surveys, such as Hemophilia Experiences, Results and Opportunities (HERO), that assessed psychosocial issues in PWH [31–33], we assessed adherence using the validated VERITAS scale and used the SF-36 (vs the EuroQoL-5 dimensions [EQ-5D]) to assess HRQoL. The SF-36 assesses domains such as vitality and social functioning, which are not included in the EQ-5D [34]. Additionally, all patients in our study self-reported for assessments of QoL, whereas parents responded on behalf of children younger than 18 years in HERO [32]. Consistent with previous research in PWH among other age groups [35], AYA PWH or VWD who reported they had ever developed an inhibitor and those who reported moderate-to-severe chronic pain also reported worse physical HRQoL in this study. Previous reports suggest this finding is likely explained by the fact that most PWH report that pain interferes with daily activities and has a negative impact on their work. In addition to physical limitations in daily activity and work, prior research has suggested an emotional impact of chronic pain on quality of life as well [36, 37]. Witkop et al. demonstrated that one third of PWH report that pain impairs the ability to form close relationships and that nearly half of young adults with a bleeding disorder report being diagnosed with anxiety or depression [37]. It has been suggested that, in addition to emphasizing prophylaxis, interventions to promote acceptance of pain and to reduce negative thoughts about pain should be utilized when approaching PWH [15].

Adolescents had higher PCS and MCS scores compared to young adults, which may be related to more frequent disease-related bleeding and greater prevalence of chronic pain as individuals managing a bleeding disorder transition from adolescence to young adulthood. Previous research has underscored that adherence to treatment regimens declines with age. Diligent factor administration by parents in younger years often gives way to less treatment regimen fidelity during young adulthood where patients often begin to assume primary responsibility for their bleeding disorder care [22, 23, 38, 39]. Further, as children transition into young adulthood, their activity level often intensifies, which could heighten the risk of bleeding.

AYA females with a bleeding disorder reported lower physical HRQoL when compared with AYA men in this study, even after adjustment for other sociodemographic and clinical factors. Reasons for this are not clear. Previous work would suggest the opposite might be true given that, compared with AYA males, female AYA were more likely to have VWD (vs hemophilia) and that VWD, generally speaking, has a lower overall burden of disease than does hemophilia and is less commonly associated with the cumulative arthropathic burden in adulthood often seen in PWH [40]. However, approximately one in four patients with VWD experience joint bleeds, which can have a negative effect on physical quality of life, with the majority of first joint bleeds occurring before age 16 [40]. In addition, previous research has suggested that physical quality of life in female VWD patients may be affected by menorrhagia, dysmenorrhagia, or pregnancy-related bleeding, which could lead to anemia, fatigue, and pain [41–43]. Thus, in AYA, it is possible that acute bleeding and dysmenorrhea often associated with female VWD patients have a more immediate deleterious effect on HRQoL than do the long-term effects (e.g., arthropathy and hepatitis C) associated with hemophilia which may have not yet fully manifested. This interaction between age, sex, bleeding disorder type, and HRQoL should be more thoroughly evaluated with future research.

AYA patients who infused clotting factor prophylactically reported significantly better mental HRQoL (MCS) compared with those who infused using on-demand regimens. However, differences in physical HRQoL (PCS) were not observed in our study, which is a surprising finding given that prophylaxis is associated with fewer joint bleeds and less pain [7], and a recent review found that joint status was associated with both social and emotional well-being [44]. One possible explanation for the findings in our study is that physical sequelae of the disease have not yet manifested in AYAs, a hypothesis also set forth by Poon et al. [20]. However, prophylactic treatment may provide mental assurance to patients that their disease is well controlled, thereby leading to improved mental HRQoL. Future studies should attempt to tease out the influence of prophylaxis, disease type and severity, and comorbid illness on HRQoL.

This study has limitations. Primarily, data are cross-sectional, thus causal inference cannot be made. Specifically, although study results support that several factors (e.g., chronic pain, inhibitor development, clotting-factor regimen type) are related to HRQoL, the directionality of this relationship cannot be confirmed. That is, though we modeled how these factors affect HRQoL, it is also possible that HRQoL instead affects a patient’s willingness and ability to adequately manage his or her bleeding disorder. This cannot be teased out in a cross-sectional study and should be examined in the future with prospective studies. A second limitation is that all data are self-reported. As such, information about blood disorder type and severity, health insurance coverage, and other demographic, clinical, and behavioral factors are not confirmed by medical record review or administrative claims data. However, by obtaining data through self-report, this study was able to collect important, reliable, and valid patient-report outcomes (PRO) data about adherence, chronic pain, and HRQoL. Patient-reported data about pain and HRQoL are considered the ‘gold standard,’ and the VERITAS scales are the only validated measures of adherence to clotting-factor treatment developed to date.

Another limitation is that the VERITAS-combined score that we reported, though statistically comprised of two validated instruments, is an experimental, non-validated measure that has only been previously reported once [25]. Most participants (68%), however, treated prophylactically, and VERITAS-Pro scores, which have been previously validated, confirmed the results of the experimental, combined score. Additionally, the SF-36, used in this study to assess HRQoL, is only validated in persons aged 14 years and older [26], whereas a small percentage of patients in our study were aged 13 years (n = 9/108, 8.3%). Further, no comparisons were made between SF-36 results in study respondents versus matched normal controls or persons living with other chronic diseases. Finally, AYA PWH and VWD were primarily recruited from large national or regional hemophilia meetings. Thus, our convenience sample of AYA PWH and VWD may not adequately represent the broader AYA PWH and VWD population who do not typically attend these meetings.

## Conclusions

Efforts should be made to prevent and manage chronic pain, which was strongly related to both physical and mental HRQoL, in the AYA PWH and VWD populations. Previous research has suggested that better adherence to prescribed treatment regimens is associated with less chronic pain [25]. Future research should explore (1) why women had lower physical HRQoL scores—even after adjustment for other sociodemographic and clinical factors, and (2) how prophylaxis may improve overall mental/emotional HRQoL in the AYA PWH and VWD populations.

## Abbreviations

AYA: Adolescent and young adult
BP: Bodily pain
CI: Confidence interval
EQ-5D: EuroQoL-5 dimensions
FPS-R: Faces Pain Scale-Revised
GH: General health
HRQoL: Health-related quality of life
IQR: Interquartile ranges
MCS: Mental Component Score
MH: Emotional well-being/mental health
PCS: Physical Component Score
PF: Physical function
PWH: Persons with hemophilia
RE: Role limitations due to emotional problems
RP: Role limitations due to physical health problems
SF: Social functioning
SF-36: 36-Item Short Form Health Survey
VT: Vitality
VWD: von Willebrand disease
